# Association between Subclinical Hypothyroidism and Incident Hypertension in Women: A Systematic Review and Meta-Analysis

**DOI:** 10.3390/jcm10153318

**Published:** 2021-07-28

**Authors:** Jean Kim, Narut Prasitlumkum, Sandeep Randhawa, Dipanjan Banerjee

**Affiliations:** 1 Department of Internal Medicine, University of Hawaii Internal Medicine Residency Program, Honolulu, HI 96813, USA; sandeepr@hawaii.edu; 2 Department of Cardiovascular Medicine, University of California-Riverside, Riverside, CA 92521, USA; narutprasitlumkum@gmail.com; 3 The Queen’s Medical Center, Queen’s Heart Institute, Honolulu, HI 96813, USA; dbanerjee@queens.org; 4 John A. Burns School of Medicine, University of Hawaii, Honolulu, HI 96813, USA

**Keywords:** subclinical hypothyroidism, thyroid, hypertension, blood pressure, females, meta-analysis

## Abstract

Subclinical hypothyroidism (SCH) has been found to be associated with an increased risk of cardiovascular diseases. However, there is no clear consensus on the relationship between SCH and hypertension (HTN). We sought to investigate the association between SCH and incident HTN in women. MEDLINE and EMBASE databases were searched for studies that reported the incidence of HTN in females with SCH versus without SCH. Pooled odds ratio (OR) and 95% confidence interval (CI) of the outcome were obtained using a random-effects model. Studies were also divided into the middle-aged (mean age < 65) and the older (mean age ≥ 65) subgroups, and a subgroup analysis was performed to examine the potential age-effect on the association between SCH and HTN. Nine studies with a total of 21,972 subjects met the inclusion criteria. SCH was found to be positively associated with HTN (OR = 1.32, 95% CI = 1.02–1.71). Such association varied depending on the age of women. In the middle-aged subgroup, SCH was more positively associated with HTN (OR = 1.64, 95% CI = 1.18–2.27), while there was no significant association in the older subgroup (OR = 0.97, 95% CI = 0.80–1.16). Our study showed that the middle-aged females with SCH had an increased risk of HTN, while there was no significant association in the older females with SCH.

## 1. Introduction

Subclinical hypothyroidism (SCH) represents an early form of thyroid dysfunction and is biochemically defined as an elevated TSH (thyroid-stimulating hormone or thyrotropin) level with a normal level of free thyroxine (FT4) within the reference range [1]. SCH can affect about 1–11% of adults depending on the cohort studied, and such wide variability in its incidence can be attributed to the environmental and ethnic differences as well as the different TSH reference ranges used in each country [2,3,4,5,6,7,8].

The cardiovascular system is one of the most important downstream targets of triiodothyronine (T3), the active cellular form of thyroid hormone. Despite its relatively benign clinical course compared to an overt hypothyroidism, SCH has been found to be associated with an increased cardiovascular risk, including coronary artery disease, myocardial infarction, stroke, and dyslipidemia [9,10,11]. However, there is no clear consensus on the relationship between SCH and hypertension (HTN), with several published studies showing a positive association between SCH and HTN [12,13,14,15,16,17] and some studies showing no association [18,19,20].

As in most thyroid diseases, it has been reported that the incidence of SCH is more common in women than in men [17,21,22,23]. Moreover, the prevalence of SCH in both genders increases with age, and 8% to 18% of adults 65 years of age or older was found to have SCH [22,24,25]. For this reason, we decided to study a female patient population and examine different age groups.

The aim of this study was to elucidate the relationship between SCH and HTN in females via meta-analysis of published cross-sectional and cohort studies. Moreover, we also divided the included studies into the middle-aged (mean age < 65) and the older (mean age ≥ 65) subgroups and sought to investigate the effect of age on the association between SCH and HTN.

## 2. Materials and Methods

### 2.1. Search Strategy

A literature search for published studies indexed in MEDLINE and EMBASE databases from inception to November 2020 was conducted using a search strategy that included the terms “subclinical hypothyroidism”, “hypertension”, and “females”. The study included patients with all disease statuses and methods of conditioning regimens. There was no restriction based on patient’s age, ethnicity, race, data sources, or study location. Review articles, case reports, letters, commentaries, abstracts, and studies in languages other than English were excluded. A manual search for additional pertinent studies using references from the retrieved articles was also completed.

### 2.2. Study Inclusion Criteria

The eligibility criteria for inclusion of studies are the following:

(1) Case-control studies, cohort studies (prospective or retrospective), and cross-sectional studies that reported the incidence of HTN in females with SCH and those without SCH.
(2) Statistics such as odds ratio or hazard ratio with 95% confidence interval and *p*-values associated with t-test, Wilcoxon test, or Kruskal–Wallis test, and sufficient raw data for these calculations had to be provided.

### 2.3. Quality Assessment of the Included Studies

Newcastle–Ottawa quality assessment scale, ranging from 0 to 9, was used to evaluate each study in three domains: recruitment and selection of the participants, similarity and comparability between the groups, and ascertainment of the outcome of interest among cohort studies [26].

### 2.4. Definition of Subclinical Hypothyroidism

SCH was defined according to its universally accepted biochemical definition of elevated TSH level with normal serum free-T4 level. The exact cutoffs for normal TSH range differed in each study based on the assays used. Table 1 shows the reference TSH levels for SCH in each study.

### 2.5. Definition of Hypertension

HTN was defined as systolic blood pressure and diastolic blood pressure of >140/90 mmHg in many included studies. The reference level of HTN varied depending on included studies. Table 1 shows the specific reference levels of blood pressure for HTN in each study.

### 2.6. Middle-Aged and Older Subgroups

A geriatric population is defined as people with chronological age of 65 years or older in most literatures, and this reference range was used to categorize the included studies into either middle-aged (mean age < 65) or older (mean age ≥ 65) subgroups. Six studies pertained to the middle-aged subgroup, and the remaining three studies were categorized into the older subgroup (Table 1).

### 2.7. Data Extraction

A standardized data collection form was used to obtain the following information from each study (Table 1): title, name of authors, year of publication, country of origin, the number of participants in the SCH group, and the control (euthyroid) group with and without HTN, the mean age in the whole population, the odds ratio (OR) and their corresponding 95% confidence intervals (CI), the mean and the reference TSH level in the SCH group, and the reference level of blood pressure for HTN.

### 2.8. Statistical Analysis

Meta-analysis of the included studies was performed to determine the pooled OR and 95% CI of the outcome. In each study, OR was given by the ratio of the odds (for the incidence of HTN) in the SCH group to the odds (for the incidence of HTN) in the control group. In the studies that did not report the values of OR and its corresponding 95% CI, the numbers of subjects for the following were provided: (a) SCH with HTN, (b) SCH without HTN, (c) controls (euthyroid) with HTN, and (d) controls without HTN (Table 1). Then, by using these provided data, OR and its corresponding 95% CI was calculated [27]. The heterogeneity of effect size estimate across the studies was quantified using the Q-statistic and the corresponding *p*-value or equivalently using the I-squared (I^2^) statistic [27]. In our study, the meta-analysis was performed using the random-effects model [27,28], and the main results were summarized in a forest plot. For the assessment of publication bias, a funnel plot was used for a visual representation, and the degree of asymmetry in the funnel plot was quantitatively evaluated via the Egger’s linear regression test [29,30]. To test the robustness of the results, we conducted a sensitivity analysis by performing meta-analyses by excluding one study at a time. Moreover, we divided the whole group into the middle-aged subgroup (with mean age < 65) and the older subgroup (with mean age ≥ 65) and performed subgroup meta-analysis to examine the potential age-effect on the association between SCH and HTN. All analyses were performed using STATA 16 software (StataCorp LLC, College Station, TX, USA).

## 3. Results

### 3.1. Study Search Results

**Figure 1:**
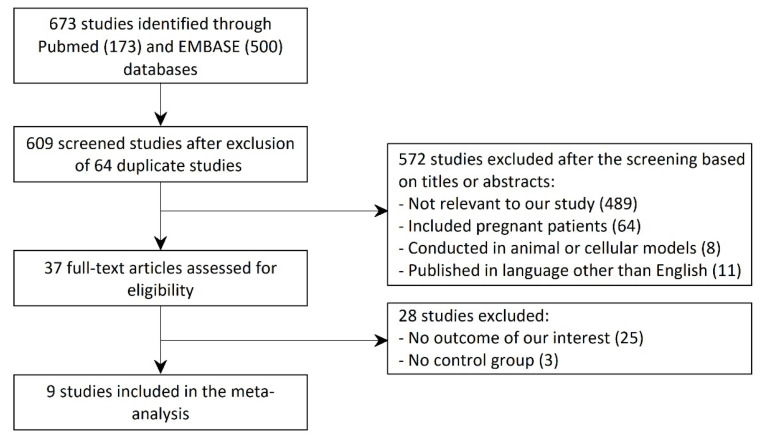
PRISMA (Preferred Reporting Items for Systematic Reviews and Meta-Analyses) flow diagram for identification, screening, eligibility, and inclusion of studies.

Figure 1 shows a PRISMA (Preferred Reporting Items for Systematic Reviews and Meta-Analyses) flow diagram that depicts the process of identification, screening, eligibility, and inclusion or exclusion of the studies. The initial search of the PubMed and the EMBASE databases yielded 673 articles (173 studies from PubMed and 500 studies from EMBASE). After exclusion of 64 duplicate studies, 609 studies underwent title and abstract review. Of these articles, 572 studies were excluded because they were not relevant to our study (*n* = 489), included pregnant patients (*n* = 64), were conducted in animal or cellular models (*n* = 8), or published in a language other than English (*n* = 11). A total of 37 articles underwent full-length review. Of these, 28 studies were excluded as they did not have the outcome of our interest (*n* = 25) or did not have a control group (*n* = 3). Thus, the final analysis included 9 unique studies with total of 21,972 female subjects.

### 3.2. Description of the Included Studies and Their Quality Assessment

The main characteristics of the included studies (*n* = 9) are described in Table 1. Comorbidities other than HTN in patients with SCH included metabolic syndrome, hyperlipidemia, and impaired fasting glucose. Regarding the study design, five studies were cross-sectional, three studies were prospective cohort, and one was a case cohort. For each study, information including the number of participants, the mean and the reference TSH level of the SCH group, and the reference blood pressure level for HTN was given.

Although the reference level of HTN was not reported in the work of LeGrys et al. [19], the number of subjects with HTN and without HTN in the SCH and control groups were clearly denoted in their study, and hence, this work was included in our meta-analysis. We also think that the given reference levels for HTN in the included studies seem to be reasonable overall, as they are in alignment with most societal guidelines for the definition of hypertension. In the study of Luboshitzky and Herer [16], the number of people with a systolic blood pressure of >140 mmHg was given as well as the number of people who had a diastolic blood pressure of >90 mmHg. For the statistical analysis, the number of people with a systolic blood pressure of >140 mmHg was used, but it should be noted that the statistical significance did not differ when the number of people with an elevated diastolic blood pressure was used instead.

Information about the mean age of the participants in each study was also recorded. Three studies pertained to the older (mean age ≥ 65) patient group, while the remaining six studies were categorized as the middle-aged (mean age < 65) group for the subgroup analysis. The average age in the older subgroup was 71.0 years old, and the mean age of the middle-aged subgroup was 49.6 years old. In the study of Zhang et al. [17], the OR and 95% CI values were given in their paper (OR = 1.959, 95% CI = 1.594–2.407). In the remaining eight studies, the number of subjects with HTN and without HTN in the SCH and control groups were given, and the OR and its corresponding 95% CI were manually calculated [27]. The Newcastle–Ottawa scale (0–9) was used to evaluate the quality of the included studies based on three domains: selection, comparability, and outcomes [26]. The range of scores for the included studies was 6 to 9, with a mean score of 7.1, reflecting a high quality of the included studies.

### 3.3. Quantitative Meta-Analysis Results

**Figure 2:**
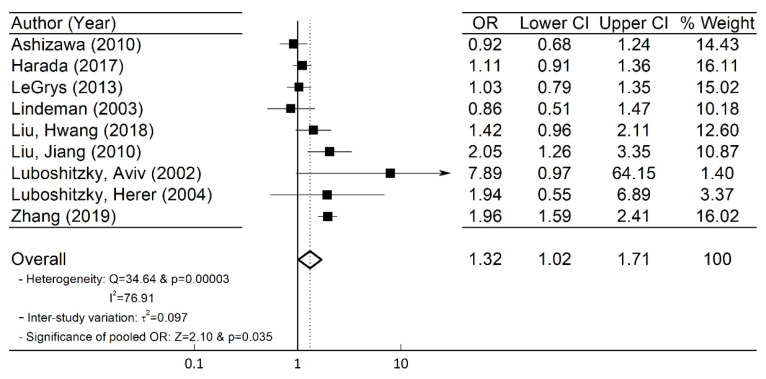
Forest plot of all included studies (*n* = 9) showing OR for the association between SCH and HTN. OR for each individual study is represented by a solid square (■), while its CI is denoted by a horizontal line. A diamond (◇) denotes the CI for the pooled OR, and the vertical dashed line passes the pooled OR. Abbreviations: OR, odds ratio; CI, confidence interval; SCH, subclinical hypothyroidism; HTN, hypertension.

A total of nine (*n* = 9) studies with 21,972 female subjects (1896 subjects with SCH) were included in our meta-analysis. Using the information in Table 1 for the number of subjects in the SCH and control groups with or without HTN, we obtained the OR and its corresponding 95% CI [27]. The forest plot in Figure 2 depicts the OR and the 95% CI of individual study. Heterogeneity among the included studies existed as the Q-statistic, and its corresponding *p*-values were 34.64 and 0.00003, respectively. We also quantified the degree of heterogeneity by using the I^2^ statistic, which indicated a high heterogeneity among the studies (I^2^ = 76.91%). Thus, in our study, we employed the random-effects model [27,28] and obtained the overall pooled OR (=1.32) and 95% CI (=1.02–1.71) as shown in Figure 2.

In comparison to the overall pooled OR and 95% CI, the work of Luboshitzky et al. [15] had a much larger OR (=7.89) and wider 95% CI (=0.97–64.15). We note that the weight for each study is related to the inverse of the variation (Var) of ln(OR) [27]; as the Var(ln(OR)) increases, the weight decreases. Since Var(ln(OR)) (=0.95715) in the work of Luboshitzky et al. [15] is the largest one in all the included studies, its normalized % weight (=1.40%) becomes the lowest one. Hence, its contribution to the pooled OR and 95% CI was properly considered in the random-effects model by considering its smallest normalized % weight. In this way, in the case of high heterogeneity, the random-effects model may be effectively employed for meta-analysis by taking into consideration the normalized % weight in each study [27].

To determine the statistical significance of the pooled OR, we obtained the Z-statistic (=2.10) and its corresponding *p*-value (=0.035), which was found to be statistically significant (*p* < 0.05). Consequently, SCH was found to be associated with an increased incidence of HTN in females (i.e., women with SCH had 32% increased odds of incidence of HTN compared to euthyroid women).

### 3.4. Publication Bias

**Figure 3:**
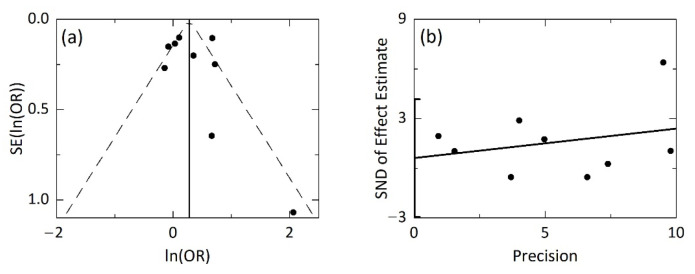
Publication bias in the whole studies (*n* = 9). (**a**) Funnel plot of (ln(OR*_i_*), SE(ln(OR*_i_*))) [denoted by solid circles(●)] for individual studies (*i* = 1, …, 9). Vertical solid line passes the pooled ln(OR) (=0.277). Left and right dashed lines represents the 95% pseudo confidence limits. (**b**) Egger’s test for asymmetry of the funnel plot. Solid circles represent (Precision, SND) for individual studies; SND = ln(OR)/SE(ln(OR)) and Precision = 1/SE(ln(OR)). A solid linear regression line is shown, and the vertical line on the SND axis represents the 95% CI for the intercept. Abbreviations: OR, odds ratio; CI, confidence interval; SE, standard error; SND, standard normal deviate.

Publication bias was assessed by constructing a funnel plot of the individual studies, which was visually symmetric (Figure 3a) [29]. The Egger’s linear regression test was also performed [30], and as shown in Figure 3b, it did not suggest a presence of publication bias, as the *p*-value (=0.693) for the intercept was larger than the significance level (=0.05). Consequently, the included studies had no publication bias.

### 3.5. Sensitivity Analysis

**Figure 4:**
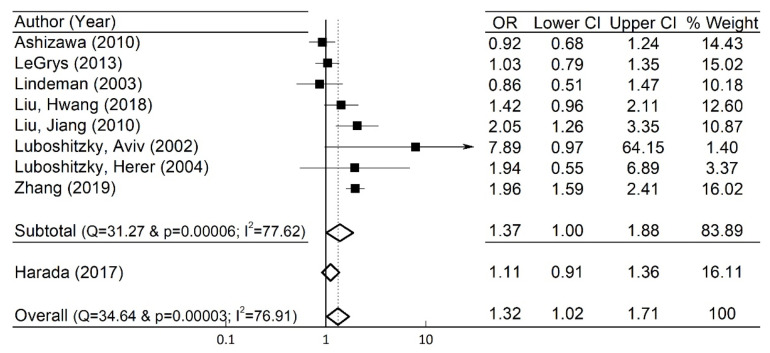
Sensitivity analysis via exclusion of the study of Harada (2017). Forest plot in a group (*n* = 8) without the study of Harada (2017). % weights are from the random-effects model in the whole studies (*n* = 9). OR for each included individual study is denoted by a solid square (■), while its CI is represented by a horizontal line. The centers and the widths of the diamonds (◇) represent the ORs and the CIs for the excluded study of Harada (2017) and the pooled ORs and CIs in the subtotal and the overall cases, respectively.

To examine the robustness of the pooled OR and 95% CI in the whole group, sensitivity analyses were undertaken by excluding an individual study at a time, and they showed no significant changes. For instance, when the study of Harada et al. [12] was arbitrarily excluded, the pooled OR (=1.37), and the 95% CI (=1.00–1.88) were close to those in the original whole group (Figure 4).

In addition to the above sensitivity analysis with the exclusion of the study by Harada et al. [12] with the highest normalized % weight (=16.11%), we also performed sensitivity analyses by excluding the works by Luboshitzky et al. [15], Liu et al. [14], and Zhang et al. [17], whose OR values were larger than the pooled OR (=1.32) in the original whole population. We found that the pooled OR and the 95% CI were 1.29 and (1.00–1.66) with the exclusion of the study by Luboshitzky et al. [15], OR = 1.25 and 95% CI (0.95–1.64) with the exclusion of the work by Liu et al. [14], and OR = 1.18 and 95% CI (0.96–1.45) when excluding the study by Zhang et al. [17], respectively. Both pooled OR and 95% CI were found to vary depending on the normalized % weights of excluded studies. In the excluded cases of the lowest % weight (=1.40%) [15] and the middle-range % weight (=10.87%) [14], their values of pooled OR were close to the pooled OR (=1.32) in the whole population. On the other hand, in the excluded case of the second highest % weight (=16.02%) [19], its deviation from the pooled OR (=1.32) in the whole population became a little larger. The sensitivity analysis with these excluded studies suggest that our results were relatively robust.

### 3.6. Subgroup Analysis

**Figure 5:**
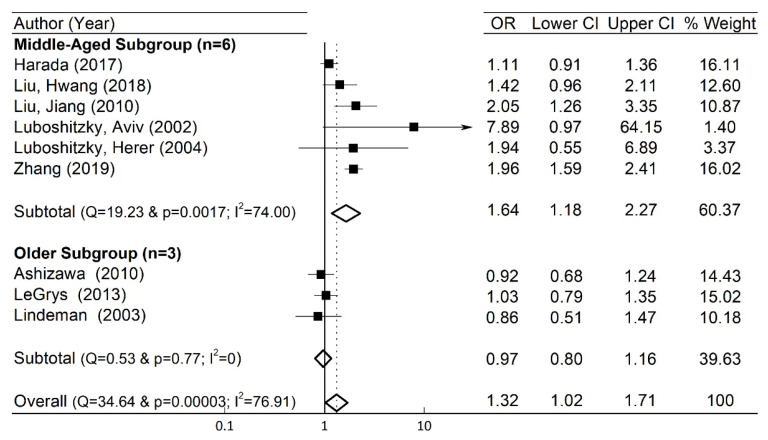
Subgroup analysis forest plots in the middle-aged (*n* = 6) and the older (*n* = 3) subgroups. % weight is from the random-effects model in the whole studies (*n* = 9). OR for each individual study in each subgroup is denoted by a solid square (■), while its CI is represented by a horizontal line. The centers and the widths of the diamonds (◇) represent the pooled OR and CI in each subtotal case and the overall case, respectively.

To assess the effect of age, a subgroup analysis was performed by categorizing the studies into either middle-aged (mean age < 65) or older (mean age ≥ 65) subgroups. The average age in the middle-aged subgroup was 49.6 years old, while the mean age of the older subgroup was 71.0 years old. The forest plot in Figure 5 summarizes our subgroup meta-analysis result. In the middle-aged subgroup with mean age < 65, SCH was associated with an increased incidence of HTN (OR = 1.64, 95% CI = 1.18–2.27, I^2^ = 74.0%) in comparison to the whole group with OR = 1.32, while there was no significant association in the older subgroup with mean age ≥ 65 (OR = 0.97, 95% CI = 0.80–1.16, I^2^ = 0.0%).

## 4. Discussion

Despite the perception of relatively benign clinical course of SCH compared to an overt hypothyroidism, SCH has been shown to be associated with an increased risk of cardiovascular diseases. In fact, it has been shown that the thyroid hormone has a profound effect on the cardiovascular system and influences the cardiac contractility, systemic vascular resistance, as well as cholesterol metabolism [9,10,11]. In this meta-analysis, we investigated the association between SCH and HTN in females. The key findings of our study include: (1) women with SCH have about 32% increased odds of incident HTN compared to euthyroid women (OR = 1.32, 95% CI = 1.02–1.71); (2) this association was found to be only significant in the middle-aged female subgroup with an average age <65 (OR = 1.64, 95% CI = 1.18–2.27) and not in the older subgroup.

First, we discuss the increased incidence of HTN in patients with SCH. Under normal physiological circumstances, thyroid hormone affects the blood pressure via its action on the ion channels, inducing endothelium-mediated nitric-oxide production and causing direct vascular smooth muscle relaxation [31,32]. Endothelial dysfunction secondary to impaired vascular smooth muscle relaxation have previously been demonstrated in SCH, which may explain the increased incidence of HTN [33,34]. We also note that thyroid hormone plays an essential role in removing excess LDL (low-density lipoprotein) cholesterol [35]. Accordingly, patients with SCH have been shown to have an increased incidence of hyperlipidemia, which likely contributes to atherosclerosis, increased arterial stiffness, and HTN [35,36].

In our study, the discrepant association of SCH and HTN in different age subgroups is notable; there was no statistically significant association between SCH and HTN in the older subgroup unlike in the middle-aged subgroup. The reason behind this discrepancy is unclear, but several suggestions can be made. Notably, the activity of type II iodothyronine deiodinase, an enzyme that converts pro-hormone thyroxine (T4) to an active thyroid hormone (T3) in target cells, has been shown to decrease with aging [25]. This in turn leads to a decreased T3 level and a reflexive increase in TSH level in older people. Indeed, in cross-sectional studies of euthyroid individuals, TSH concentrations have been shown to increase with age [37,38,39]. Moreover, several cohort studies have shown that the age-associated increase in TSH concentrations did not cause a decrease in free T4, suggesting a change in TSH set-point with aging [40,41]. Hence, age-related TSH elevation in the older subgroup may be more representative of a physiological aging process than a pathologic condition. In a randomized controlled trial for thyroid hormone replacement for untreated older adults with SCH, it was shown that the levothyroxine therapy in the elderly patients diagnosed with SCH provided no symptomatic benefit [42]. Moreover, it has been shown that elderly patients diagnosed with SCH under current guidelines do not strongly express the clinical signs of hypothyroidism compared to younger SCH patients, and this may further support the inadequacy of using the same guidelines for diagnosing SCH in the elderly population [8]. Ultimately, we must be cautious when diagnosing and treating SCH in older patients, and a guideline for age-based reference range of TSH is needed.

We also reference a previous work that investigated the association between SCH and the blood pressure [43]. This study was a meta-analysis that aimed to obtain the pooled weighted mean difference (WMD) of blood pressure in SCH versus the euthyroid groups. In contrast to our work, the subjects in the study consisted of both males and females. In this work, SCH was found to be associated with a slightly higher systolic blood pressure (SBP) than the euthyroid group (pooled WMD of SBP = 1.47 mmHg, 95% CI = 0.54–2.39, *p* = 0.002), while there was no statistically significant difference in diastolic blood pressure (DBP) between the SCH and the euthyroid groups. Moreover, a meta-regression analysis showed a significant linear relationship between age difference and WMD of SBP in the SCH and euthyroid groups. Thus, the age difference between the two groups could be a key confounding factor for WMD of SBP. Accordingly, it was concluded that SCH was associated with a slightly higher SBP, which could be attributed to the age difference between the SCH and euthyroid groups. It is important to note that the meta-analysis investigated the relationship between SCH and the mean values of SBP and DBP, but the blood pressures were not necessarily in the hypertensive range. In contrast, our paper studied the association of SCH and the incidence of HTN and a pathologic increase in blood pressures and assessed the pooled OR for the incidence of HTN. To our best knowledge, there is no published meta-analysis that studied the association between SCH and HTN.

Finally, we discuss relevant limitations in our study. In the present study, we focused on the association between SCH and HTN in women, as the incidence of SCH is more common in women. However, in future studies, it would be interesting to examine whether the same association between SCH and HTN is present in the male population also. Secondly, since all the included studies are observational, the meta-analysis might be affected by confounding factors, and hence, the results must be carefully interpreted even though they may provide useful information. Secondly, owing to our inclusion criteria, publication bias may not be completely excluded, as unpublished studies were not included. Thirdly, the TSH cut-off reference level for the SCH and the reference level of HTN varied depending on included studies, which might also affect overall interpretation. Nevertheless, our sensitivity analysis showed unaltered outcomes, which suggested that the overall analysis is robust. Lastly, most of the included studies (except for the work of LeGrys et al. [19], with minimum five-year follow-up) measured their data points for TSH and blood pressures at a single time point, which may lead to less accurate and robust diagnoses of SCH and HTN. In the future, observational studies with longer follow-up periods are needed to establish stronger evidence for the cause and effect relationship between SCH and HTN.

## 5. Conclusions

In our meta-analysis of nine studies, SCH was found to be associated with an increased incidence of HTN in women (OR = 1.32, 95% CI = 1.02–1.71). Specifically, the females with SCH in the middle-aged subgroup with mean age < 65 had an increased risk of HTN (OR = 1.64, 95% CI = 1.18–2.27), while there was no significant association in the older subgroup with mean age ≥65 (OR = 0.97, 95% CI = 0.80–1.16). To confirm this conclusion, a well-designed, large-scale prospective cohort study including all age subgroups is needed. In the case of the middle-aged females with SCH, careful monitoring of cardiovascular disease may be warranted. Societal guidelines suggest that subclinical hypothyroidism is not recommended to be treated unless TSH level exceeds 10 mIU/L. However, based on our result that showed the positive association between SCH and HTN in middle-aged women, treating SCH in this patient population when signs of rising blood pressures are observed, regardless of patients’ TSH levels, should be considered. On the other hand, in the older patients with SCH, the elevation in TSH levels could be due to a natural aging process, and routine thyroid replacement therapy may not result in predictable benefits; therefore, treating SCH in this subgroup should be done with caution.

## Figures and Tables

**Table 1:** Main characteristics of the included studies (*n* = 9). Three studies with the superscript “^°^” belong to the older subgroup with mean age ≥ 65.

Author	Country	Published Year	Study Type	SCH (HTN(a)/ No HTN(b)) (1896)	Control (HTN(c)/ No HTN(d)) (20,076)	Mean Age	Odds Ratio (95% CI)	Mean (Reference) TSH Level in SCH (mIU/L)	HTN SBP/DBP (mmHg)
Ashizawa °	Japan	2010	Cross- Sectional	194 (110/84)	2134 (1253/881)	71.5	NR	5.98 (>4.5)	>140/90
Harada	U.S.	2017	Prospective Cohort	573 (167/406)	2571 (694/1877)	56.9	NR	NR (>4.2)	>130/85
Legrys °	U.S.	2013	Case Cohort	282 (85/197)	3381 (995/2386)	67.5	NR	5.85 (>4.68)	NR
Lindeman °	U.S.	2003	Cross- Sectional	74 (27/47)	283 (113/170)	73.9	NR	NR (>4.7)	>160/95
Liu, Hwang	Taiwan	2018	Cross- Sectional	102 (45/57)	6323 (2257/4066)	48.5	NR	NR (>5.5)	>130/85
Liu, Jiang	China	2010	Cross- Sectional	75 (31/44)	724 (185/539)	44.8	NR	6.8 (>4.8)	>140/90
Luboshitzky Aviv	Israel	2002	Prospective Cohort	57 (11/46)	34 (1/33)	46.8	NR	10 (>4.5)	>140/90
Luboshitzky Herer	Israel	2004	Prospective Cohort	44 (15/29)	9 (4/15)	51.6	NR	9.2 (>4.5)	>140
Zhang	China	2019	Cross- Sectional	495	4607	48.8	1.959 (1.594, 2.407)	NR (>5.0)	>140/90

Abbreviations: SCH, subclinical hypothyroidism; TSH, thyroid-stimulating hormone; HTN, hypertension; SBP, systolic blood pressure; DBP, diastolic blood pressure; NR, not reported.

## Data Availability

The datasets generated and analyzed during the current study are available from the corresponding author on reasonable request.
